# Mr. Harrold, in Answer to Dr. Kinglake

**Published:** 1806-08

**Authors:** E. Harrold

**Affiliations:** Cheshunt, Herts


### To the Editors of the Medical and Phyſical Journal.

GENTLEMEN,

I Will neither amuse nor tire your readers by a reiteration of complimentary language. I shall however, just remark, that it is extremely pleasant, in controversial matters, where there ought to be as little personality as possible, to see men write temperately, and address each other in the language, and with the consideration of Gentlemen. It certainly has great influence in forwarding the cause of truth, and induces many to enter the lists, who, though not very deficient in talent, or in the power of communicating information, might otherwise be deterred by the want of courage.

Analogy, though a very useful, is not always a safe guide in our reasoning. Dr. Reid has well remarked, that "Arguments from analogy are always at hand, and grow up spontaneously in a fruitful imagination; while arguments that are more direct, and more conclusive, often require painful attention and application: and therefore, mankind in general have been very much disposed to trust to the former."* I do not think that much is to be gained by pursuing a *false* analogy: I consider Dr. Kinglake's to be such; and as his authority may give it some influence, it may be useful to prove its fallacy. In speaking of what, he is pleased to say, I have "unconsciously conceded," he has not made any distinction between *the powers of the mind*, and the *vital principle*; and the want of this distinction seems to be the foundation of *his* error. That "no theory of animated nature can dispense with this sort of connection" is true, as far as it relates to *matter and life*; but that it is so with respect to "matter and mind," I must have liberty to deny, unless Dr. K. speaks of the divine mind. For, notwithstanding the poetical fiction of the "Loves of the plants," Dr. K. will hardly admit of the *mental* energy of the vegetable kingdom, though it possess life and an organization, subject to the influence of external agents; for my own part, I should as soon believe in "the Loves of the Triangles." The act of locomotion, arising from the influence of volition upon the locomotive powers, is surely very different from

---

* Inquiry into the human mind.

involuntary organic action; the latter being excited by mechanical or chemical stimulus upon the vital principle, the former by motive operating on the mind.

But to return to the analogy itself. Suppose Dr. K. spurring his horse, and the reluctant animal, by this stimulus to his voluntary efforts, proceeding at the rate of twenty miles per hour; so long as the spur is used with sufficient vigour, and the powers of the animal are unexhausted, he may proceed at the same rate; if he be spurred *less* severely, he will slacken his pace; but if the severe spurring be suddenly discontinued, he will be very likely as suddenly to alter his pace from a gallop of twenty miles per hour, to a trot of six or seven; and in that case Dr. K. must have a strong seat or be thrown out of his saddle, or, perhaps, precipitated over his horse's head; a situation in which I should be very sorry to see him.

This subject might be pursued a great deal further, and the arguments of the gravest metaphysicians might be employed in its support; but they would probably be as useless as numberless volumes on metaphysics were before the introduction of the principles of inductive philosophy. My object is to shew the necessity of caution in the use of analogy, and the danger of *false* analogy.

Dr. K. and I differ most essentially in our opinions of the "exciting cause" of inflammation in burns, which he states to be "Spirit of Turpentine" but which I take to be fire.

Dr. K. observes, that "the influence of excessive cold, on the living animal fibre, reduces the active powers of life to a state bordering on death; such a state of vital power is too feeble to admit of *violent excitement*, without risking a mortal exhaustion of its sinking remains." But violent excitement is here a relative term, for the ordinary exciting powers would in this state produce violent excitement, and therefore exciting powers far below the ordinary standard are proper. On the contrary, after the influence of fire; or excessive heat, "on the living animal fibre," the terebinthinate remedy, though *above* the ordinary exciting powers, does not augment, but diminishes the excitement, and occasions no pain. But the application of any very low degree of heat, (if the injury be such as to render the reproduction of parts necessary) will occasion such a degree of indirect debility in the producing parts, as almost to disqualify them for that office; and, when they do act, their action is extremely irregular, affording an organization extremely weak, or so exuberant as, as to be restrained with difficulty, and ultimately occasioning a very disfiguring cicatrization.—In favour of the terebinthinate remedy, it is to be observed, that its use is less troublesome to all parties, and not likely to cause either catarrh or rheumatism; consequences of the cold applications, on some occasions, too serious to be disregarded.

Dr. K. afterwards says, "that *whatever* may have occasioned a departure from the standard of health, whether such departure should consist in an *excessive* or *deficient* action of vital power, a *prompt* restoration of that standard is the eligible mode of cure." Forgetting probably what he had said in the preceding page, that "a *slow* augmentation of exciting power in such circumstances (the effects of excessive cold) is most safe, and best adapted to restore and establish the energies of life." This is all the concession that *I* wanted.

The distinction is surely *not* without a difference between the application of fire, only for a sufficient time to produce inflammation, and its *continued* application; between the inflammation arising from an irritating matter *withdrawn*, and the inflammation arising from irritating matter *still in contact* with the inflamed part.

Having thus, Gentlemen, explained myself, as far as to me appears necessary, I shall leave the subject in abler hands, at least till farther experience either confirms, or makes me change my present opinion. I have no doubt of the facts advanced by Dr. K. I am confident in my own,—it is therefore at *least* a matter of very curious inquiry. In common with most men, I naturally like to feel that I am right, but I am not anxious to support any opinion that time and farther experience may not warrant, and shall always be happy to meet with the liberal and gentlemanly criticism that I have encountered on the present occasion. I am, &c.

E. HARROLD.

*Cheshunt, Herts. July* 3, 1806.

